# Animal Biology

**Published:** 1887-12

**Authors:** 


*Animal Biology*. By C. LLOYD MORGAN. Pp. 370. London: RIVINGTONS. 1887.

This work is intended as an elementary text-book, suitable for young men intending to present themselves for the London Intermediate and Preliminary Scientific Examinations, and for other similar examinations such as a medical student may be called upon to pass. For this purpose the work is admirably suited—we know of none better. The course presented in the volume is a thoroughly practical one. Certain creatures, easily procurable, are taken as types, and from these, by successive stages, are evolved lessons on the Anatomy, Physiology, Histology, and Embryology of Vertebrate and Invertebrate Animals. Every step in instruction is taken with the dissection in front of the student; and every lesson is elucidated by original and most suggestive sketches by the author. The whole work is most admirably planned and most ably carried out. The clear, simple language, and pleasant fluent style, add greatly to the charm of the book, which is certain to be a favourite with medical students.

